# Theorizing “Place” in Aging in Place: The Need for Territorial and Relational Perspectives

**DOI:** 10.1093/geront/gnad002

**Published:** 2023-01-19

**Authors:** Sophie Yarker, Patty Doran, Tine Buffel

**Affiliations:** Department of Sociology, University of Manchester, Manchester, UK; Department of Sociology, University of Manchester, Manchester, UK; Department of Sociology, University of Manchester, Manchester, UK

**Keywords:** Age-friendly environments, Conceptual development, Inequalities, Place attachment

## Abstract

This paper argues for a greater theorization of “place” within aging-in-place research. It extends calls for a relational conceptualization of place by demonstrating the need for aging-in-place researchers to also pay greater attention to territorial aspects of place. This complementary understanding will help establish a new spatial grammar within aging-in-place research, that not only would improve conceptual clarity to aging in place, but would also support a more critical engagement of aging in place in questions of inequality. The paper demonstrates this through a discussion of 2 forms of inequality pertinent to older people: the uneven capacity of places to support older people and experiences of social exclusion in relation to place attachment for older people from marginalized groups.

Aging in place has become an important policy response to population aging primarily focused on supporting people to remain in their chosen homes and communities as they age. Despite important advances over recent decades, there has been some criticism that the concept remains largely “ambiguous and uncritical” (Finlay et al., 2021, p. 224), with a call for greater engagement with questions of inequality. In response, this paper argues that greater conceptual sharpness might be gained through a more thorough theorization of “place” in aging in place. Not only would this improve conceptual clarity to aging in place, but it would also support a more critical engagement of the concept in questions of inequality.

Aging in place is often defined in contrast to aging in an institutional setting or an assisted living environment, emphasizing the importance of maintaining a degree of independence. For example, Horner and Boldy (2008) define aging in place as a “positive approach to meeting the needs of the older person, supporting them to live independently or with some assistance for as long as is possible” (p. 356). However, as the field has evolved, scholars have increasingly recognized the equal importance of sociality or community. This emphasis on connection and relationships is reflected in the World Health Organization’s (WHO) definition of aging in place as “to remain at home in their familiar surroundings and maintain the relationships that are important to them” (WHO, 2020, p. 37). Social connectedness is also recognized as a key dimension in the WHO’s framework of age-friendly cities and communities (2007), which underpins the drive toward aging in the “right” place, highlighting not only the importance of community resources and assets that enable full participation in later life, but also the need for understanding aging in place as a *process.* Following this, Rogers et al. (2020) suggest that aging in place should be defined as “one’s journey to maintain independence in one’s place of residence as well as to participate in one’s community” (p. 9).

Issues about home, place, and the environment have emerged as influential themes in the study of aging. They have been especially important in the development of the subdiscipline of environmental gerontology, which emerged through the work of Lawton (1982) who examined the reasons why some physical contexts achieved a better fit with the needs and abilities of older residents than others. Other theoretical approaches have focused on the experiential dimensions of aging in place. Rowles (1983), for example, developed the concept of place attachment by drawing upon phenomenological approaches. His research demonstrated how older people who have resided in the same community for a long period of time develop a sense of attachment through a lifelong accumulation of experiences in a place, which can provide a sense of identity. Such issues of identity, familiarity, and attachment to place are also central in studies focusing on how the domestic environment meets the needs of individuals, influencing choices between moving and making alternations to the domestic space (Boldy et al., 2011; Wiles et al., 2012).

The concept of aging in place has also been expanded to consider the role that the wider neighborhood environment can play, as well as the social environment of a place (Pani-Harreman et al., 2021). This can be crucial in understanding why a person may want to remain in their neighborhood or to move. Versey (2018), for example, found that some older people living in Harlem, NY, valued staying in a neighborhood where they could remain part of social networks with neighbors and friends above moving to be closer to family, despite changes in the physical environment of their neighborhood due to processes of urban change. This means that there are functional aspects of an environment that may support well-being of older people as well as emotional aspects, such as a sense of belonging and attachment and the importance of memory and biography in place.

There has also been recognition of the multiplicity of *places* that are important to aging in place, with studies showing that aging in place does not always have to mean remaining in, or having a sense of attachment to, only *one* place (Buffel, 2015). The latter represents relational theories of aging in place, which have been used to challenge ideas of place as bounded and static (Massey, 1991), suggesting, instead, that places are made through interactions and are always developing and changing over time (Finlay & Finn, 2021). Here, a more dynamic understanding of both place and aging in place begins to emerge as do questions such as what does it mean to age in the right place (Golant, 2015) and what happens when places change.

Despite these advances in the study of aging in place, several limitations can be identified. First, there has been a lack of recognition of the unequal capacity of places to support aging in place (Cribbin et al., 2021). For example, housing and neighborhoods chosen earlier in people’s lives may no longer be appropriate to meet their needs (Golant, 2009) plus places themselves change through processes such as economic decline or regeneration, meaning that some places present more hostile and challenging environments for aging in place (Lewis & Buffel, 2020). Related to this, not all places have equal capacity to support the effective administering of aging-in-place or age-friendly policies (Yarker & Buffel, 2022).

Second, there is a lack of recognition of the types of social exclusion experienced by some members of the older population. The treatment of “older people” as a homogenous category fails to adequately recognize the diversity of needs (Wiles et al., 2012). Finlay et al. note that although aging-in-place policy routinely acknowledges cultural diversity, this is rarely put into practice, and even less attention is paid to forms of structural disadvantage. Finlay and Finn (2021), for example, have criticized age-friendly housing developments in U.S. cities for only catering to the “healthy and wealthy,” thereby reproducing housing inequality. Byrnes (2011) suggests that in the United States, the adoption of aging-in-place policy is based on an understanding of relative privilege and affluence and therefore may not meet the needs of older people experiencing different forms of social exclusion.

Finally, although environmental gerontology has made the role of place in shaping the experience of aging explicit, Andrews et al. (2013) argue that aging-in-place research is often done without an adequate theorization of *place* as a concept in itself. Despite some exceptions (see Milligan & Wiles, 2010; Peace, 2023; Peace et al., 2006), Andrew et al. make the point that space and place are left largely conceptualized as bounded, static, and relatively abstract. Addressing this need for theoretical depth in the field, the authors advocate for a greater engagement with relational theories that conceptualize place through interactions and relations which are always developing and changing over time (Massey, 2005). In this view, aging in place is a process which “recognises that the individual experience of place is layered and that knowledge of personal biography and experience in time and space leads to greater understanding of the complexity of person-environment interaction” (Peace et al., 2011, p. 754).

The gaps in our current understanding of aging in place identified here suggest a blind spot in terms of how different forms of inequality can affect the experience of aging in place. The inadequate theorization of place identified by Andrews et al. adds to the difficulty of being able to critically engage with questions of different forms of inequality. Therefore, to strengthen the study of aging in place, we “must examine current theories so that they can speak more fully about the thoughts and experiences of … disadvantaged groups” (Byrnes, 2011, p. 261). This requires further engagement with critical understandings of urban change, social exclusion, and structural inequalities (Buffel & Phillipson, 2016). This paper suggests that theorizing place through both its territorial and relational aspects provides one route toward achieving this.

Whilst supporting a greater engagement with relational theories of place, this paper develops and extends Andrew et al.’s argument to show how *territorial* approaches to place can be used to compliment *relational* ones to strengthen research on aging in place. Drawing on debates around the conceptualizing of place from human geography, the following section provides a brief outline of territorial and relational approaches to place, drawing attention to the importance of both *connections* (emphasized by relational approaches) and *boundaries and borders* (emphasized by territorial approaches) to how we understand *place* in aging-in-place research. Following this, the paper demonstrates how adopting both a relational *and* territorial approach in aging in place can support the development of a more critical perspective within the literature and allow for different questions around inequality to be brought into focus. This allows for a critical study of inequalities within aging-in-place research.

## Toward a Territorial *and* Relational Conceptualization of *Place* in Aging in Place

This paper builds on Andrews et al.’s (2013) call for a relational conceptualization of place in aging-in-place research. It argues that an acknowledgment of both the *territorial* aspects of place (the role of geographical boundaries and borders) and the *relational* aspects of place (the networks and connections within and between places) would increase the conceptual sharpness of aging-in-place research. Thus, to avoid a binary understanding of place as either territorial *or* relational, we argue that “place” in aging-in-place research should be viewed “not as either/or choices but from a both/and perspective” (Pike, 2007, p. 1147), reflecting an understanding of place as simultaneously localized and global, and as both fixed and mobile. In other words, privileging neither boundaries nor connection (territorial nor relational) but recognizing the interdependency between the two. In this section, we first demonstrate the importance of engaging with *relational* theories of place in aging research, and then continue to argue how a *territorial* approach can further deepen our understanding of aging in place.

Relational theories of place draw on a broader relational turn in the social sciences that emphasizes the relationships and connections of places. A neighborhood should not be viewed as a discrete entity; rather, places have become “unbound” or opened up by the forces of globalization (Amin, 2004). Therefore, to understand a place, it must be viewed through its connections with others, as being constituted through “a kaleidoscopic web of networks and relational connections which are not fixed or located in place but are constituted through various circulating entities” (MacLeod & Jones, 2007, p. 1179). These “various circulating entities” can be understood as the forces of globalization referred to by Amin, that is, the circulation of “goods, technologies, knowledge, people, finance, and information” (Massey et al., 2003, p. 25).

In providing an example of how this conceptualization of place would be applied to aging-in-place research, Andrews et al. (2013) consider how it might be applied to the study of age-friendly cities. They demonstrate how a relational perspective allows researchers to focus on how different places across the world are connected through the mobility of age-friendly policy, following the inauguration by the WHO (in 2010) of the Global Network of Age Friendly Cities and Communities. The result has been interventions in areas such as housing in later life, transportation, outdoor spaces, and social participation (Buffel & Phillipson, 2019). The WHO framework has been adopted by over 1,300 cities and communities and has become embedded in local places through “processes of local experimentation and implementation” (Andrews et al., 2013, p. 1360). Conceptualizing place relationally then allows aging-in-place researchers a greater insight into the mobility of age-friendly policies, by illuminating how policies developed by one institution, in this instance, by the WHO, have different expressions depending upon the particular national contexts they are embedded within.

This paper develops the argument that to advance the conceptualization of *place* in aging-in-place research, we also need to engage with *territorial* theories. Indeed, we would caution against overstating the relational aspects of place at the expense of its territorial aspects (see also MacLeod & Jones, 2007; Morgan, 2007; Pike, 2007) because a relational conceptualization of place, which due to its emphasis on mobility and flow can sometimes appear as rootless and without boundaries, is not always the most appropriate one to enhance our understanding of the meaning of places as they are lived and experienced. For example, questions about how older people develop territorial identity, a sense of home, or attachment to place require a territorial perspective, including boundaries—real or imagined—around particular localities (Yarker, 2018).

Therefore, aging-in-place research needs to recognize the continued importance of territory to conceptualizing place as well as its relational aspects. In doing so, this paper builds on established traditions within social gerontology that take a similarly balanced approach toward understanding place, yet which may not explicitly engage with the same spatial grammar. Life-course perspective, as developed by Glen Elder (1998), suggests that a holistic and dynamic understanding of person–environment relationships can only be achieved by examining the historical, geographical, and socioeconomic contexts influencing people’s life course (Chaudhury & Oswald, 2019). The life-course perspective can be visualized as a dynamic social ecological model within which micro (individual), meso (institutional), and macro (societal) influences are seen to affect development over time. This dynamic understanding of different processes, contexts, and geographical scales has been drawn upon by researchers in social gerontology to show how factors associated with place can influence life outcomes and behaviors. For example, in their longitudinal analysis, Lewis and Buffel (2020) identified how aging in place is affected by both changing life-course circumstances and the dynamics of neighborhood change over time, such as demographic change, regenerated urban spaces, and changes in neighborhood infrastructure.

A key argument of this paper is that using both a territorial and relational approach in aging in place allows for questions of different forms of inequality to come into focus. This will be demonstrated by this paper in two main ways: (1) the ability to theorize the unequal capacity of places to support aging in place and (2) the potential for understanding how different groups of older people experience social exclusion from certain environments.

### The Unequal Capacity to Support Aging in Place

Viewing aging in place through a territorial and relational lens can help reveal the unequal capacity of neighborhoods to support aging in place. Scholars of urban aging, in particular, have drawn attention to the uneven capacity of different neighborhoods to support aging in place for different groups (Lewis & Buffel, 2020). This can be for several reasons; for example, urban processes such as the privatization of public space or gentrification can result in older people living on low incomes being physically or symbolically excluded from spaces in their neighborhoods as they are redesigned to meet the needs of more affluent social groups. This can, therefore, leave some places unable to support the social connections of their existing aging populations. For example, Buffel and Phillipson (2019), in their study of aging in place in gentrified neighborhoods, discuss how some long-term older residents avoided some spaces and appropriated others differently because of feeling excluded from some of the new amenities that had opened as a result of gentrification.

Equally, austerity measures can lead to the closure of vital spaces of social infrastructure, such as libraries or community centers, and a hollowing out of the social and physical environment needed to support aging in place (Buffel & Phillipson, 2019; Finlay et al., 2021; Yarker, 2022). Libraries, community centers, green and public spaces, voluntary organizations, local post offices, and shops are a vital part of the social infrastructure of neighborhoods, and often act as places of refuge for vulnerable populations such as homeless people, people with mental health issues, recent immigrants, and some younger and older people. Research in the United Kingdom has shown that although the closure of social infrastructure and third spaces negatively affect the well-being and quality of life of older residents, those with minoritized and marginalized identities, such as older people on low incomes, ethnic minority older people, those with disabilities, long-term illnesses, and cognitive impairments, are particularly affected (Buffel et al., 2021; Yarker, 2022).

The implications of this are that some groups of older people living in underresourced neighborhoods are left feeling trapped in place (Buffel et al., 2021) or “feeling out of place” (Philips et al., 2011). Such experiences can be amplified for older people with further marginalized identities. For example, Chatters et al. (2020) describe the double jeopardy of agism and racism faced by Black adults living in low-income neighborhoods in the United States and the detrimental health impacts of this, whereas older people living with disabilities and long-term illnesses are often cut off from vital support services (AGE Platform Europe, 2020).

The drive to understand ways to enhance the capacity of neighborhoods to support aging in place has led gerontologists to study a variety of different forms of community. For example, the Village model, developed most extensively in the United States, involves older residents working together to form membership-based groups to address a variety of age-related needs. Villages in this context are defined as “self-governing grassroots, community-based organizations developed with the sole purpose of enabling people to remain in their own homes as they age” (Graham et al., 2014). Therefore, the Village model supports aging in place through the pooling of resources in a geographical location; however, it has been noted that participation in the Village model is restricted for older adults with more health needs or fewer financial resources (Lehning et al., 2017), and therefore runs the risk of further exacerbating the unequal capacity of place.

Understanding the unequal capacity of neighborhoods to support aging in place requires an understanding of locality and how localities change, therefore recognizing that a place’s capacity to support aging in place can change also (Lewis & Buffel, 2020). Thus, to fully grasp the *capacity* of neighborhoods and different forms of community initiatives to support aging in place, we require an understanding of both *territorial* and *relational* aspects of place, as well as the *interdependencies* between these two. This is because the barriers to aging in place in a particular locality (or territory), such as the lack of age-friendly facilities (benches, green spaces, opportunities for civic and social participation), can only be understood in relation to broader structural forces such as those associated with urban development, austerity, and privatization. The argument for a territorial perspective here focuses on its ability to illuminate the impact of processes of social change as experienced by those aging in place. Put another way, it provides opportunities to reveal the *local expression of macro-social forces* and their impact on the capacity of places to support older people in their everyday activities as they are lived out and experienced in the locality. This is essential to driving forward a research agenda on how different forms of community can support aging in place.

### Experiences of Exclusion From Aging in Place

When viewed through both a relational and territorial lens, different forms of social exclusion from aging in place are brought into focus, particularly for marginalized groups. The ability to age in place can be compromised by experiences of social exclusion and a critical approach to aging-in-place research must seek to understand such experiences. The concept of place attachment has provided an important way of studying social exclusion in gerontology. This section of the paper will demonstrate how a territorial and relational lens to aging in place can help illuminate different forms of place attachment for older people, and how this can be used to understand experiences of exclusion for different groups.

Place attachment is understood as the affective, cognitive, and behavioral ties that individuals develop with their environment (Woolrych et al., 2022). Relational theories of place have been instructive in ensuring place attachments are not thought of as static, but as a negotiated and constructed state through ongoing interactions between the individual and their environment. Therefore, attachment to place is shaped by continually reintegrating with places and renegotiating meanings and identity (Wiles et al., 2012). This relational view of place attachment is useful as it recognizes aging in place as being contingent on multiple places and relationships, all of which are dynamic and interconnected.

However, it is important not to overstate mobility and flow in place attachment at the expense of roots and rootedness. Territorial aspects of place are important here too especially in appreciating how boundaries and borders are used by older people to make sense of place. One of the most influential uses of the concept of place attachment within aging-in-place research comes from Rowles’ (1980) identification of physical, social, and autobiographical “insideness” (drawing on Relph, 1976) used to describe feelings of attachment and involvement in place across the life course. To feel a sense of “insideness” depends upon an awareness of boundaries (real or imagined) between what is considered “inside” or otherwise.

Recent studies of home within social gerontology have challenged static notions of the concept and instead drawn attention to “the interrelationship between the dwelling and its surrounding material, social and relational environment” (Webber et al., 2022, p. 2). Webber et al. (2022) argue that feelings of home in later life are not fixed, but subject to being made and unmade over time due to changes in the local environment and the weakening (or disappearance) of social connections. This dynamic relationship between home and how it connects (or otherwise) to the wider neighborhood elegantly demonstrates the need for understanding the role of both connections and boundaries in place.

The experience of foreign-born migrants also helps illuminate the role of both boundaries and connections in aging in place. There is a limited but growing body of research into the experience of aging in place for migrants, and specifically how people aging in places other than their country of origin negotiate attachments to place (Ryan et al., 2020). Ryan et al. (2020) describe the need to understand the attachment to place for older migrants as a continual process of constructing place identities that require “effort negotiation and adaptation over time” (p. 2). This means an experience of aging in place which is experienced at multiple scales and involves a process of embedding (and disembedding) in place (Ryan et al., 2020). This process of embedding, Ryan et al. argue, is “strongly associated with relationality and networks of family and friends” (p. 6) but also cannot be looked at only in relation to one place, “their lives are shaped by relationships, people and places both in the country where they are growing older, and elsewhere” (Zontini, 2015, p. 328). This also demonstrates the need for an understanding of transnational networks of support in aging-in-place research.

Here, a relational and territorial theorization of place allows us to understand older people’s support networks as “mobile, spawning communities of relational connectivity that transcend territorial boundaries” (Morgan, 2007, p. 33), yet which remain deeply local in character as they are developed, negotiated, and experienced in local neighborhoods. Similar to discussions of how older people make and unmake connections between the home and the wider neighborhood (see Webber et al., 2022), the negotiations of attachments to multiple places in later life demonstrated by migrants illustrated the role both boundaries and connections play within aging-in-place research.

This section has demonstrated the need to include territorial perspectives of place alongside relational ones in aging-in-place research, and how such conceptualizations have the potential to bring new insights into the different types of inequality associated with aging in place. Viewing place attachment and understandings of home through a territorial and relational lens draws attention to the complex forms of both “insideness” and exclusion from place experienced by older people. This is especially important for recognizing experiences of aging in place for marginalized groups. The struggles older migrants may have in forming attachments to places that are not able to meet their practical, social, or cultural needs, can lead to people feeling “out of place” (Cuba & Hummon, 1993) and at risk of social exclusion. Similarly, Lewis and Buffel (2020) argue the ability for some older people living in economically marginalized or rapidly gentrifying neighborhoods may have their attachments undermined. This can lead to social exclusion and other forms of inequality.

## Conclusion

This paper has suggested that a deeper conceptualization of *place* in aging-in-place research is needed to allow researchers to further their critical engagement with how different forms of inequality affect aging populations. To do this, the paper has presented a spatial grammar that acknowledges the conceptual vantage points of both relational and territorial aspects of place. Such a complementary understanding of place, we argue, provides the tools to understand how the places in which we age are both shaped by their position within wider networks of power relations, but also deeply embedded in the particularity of local context and territorial politics.

A relational and territorial conceptualization of place is particularly important for research aimed at understanding how inequalities shape the experience of aging in place. This conceptualization results in an increasingly clear division between those able to identify with particular locations that are viewed as affirmative of their own biographies and experiences, and those who experience rejection or exclusion or who see the macro-social changes affecting their neighborhood as incompatible with their ideal of aging in place (Phillipson, 2007). In this context, relational conceptualizations of place are especially helpful in illuminating how the experience of aging in place is shaped through unequal power dynamics arising from macro-social forces and trans-local connections with cities and institutions scattered around the world. At the same time, territorial perspectives allow us to *locate* the asymmetrical geometries of power and to reveal the *local expressions of macro-social* forces and their impact on the *unequal capacity of places* to support people to age in place. Territorial aspects of place, such as borders and boundaries, also give us a perspective from which to understand place attachment in later life. They allow aging-in-place researchers insight into how older people live their lives in local places, how they attach memories to them, and how they draw imaginary boundaries around places to distinguish between places that *support* them in later life and those that *exclude*.

This is a spatial grammar that is able to center inequalities, allowing new questions to emerge within the aging-in-place agenda, questions that connect to wider concerns of social and spatial justice (Greenfield, 2018). This has important consequences for the application of aging in place through age-friendly agendas. One outcome might be that it allows for a discussion of more radical age-friendly approaches. This would support the work of scholars such as Finlay et al. (2021), who caution against the uncritical celebratory approaches to age-friendly communities and encourage seeking approaches that might challenge capitalist or neoliberal ideologies in cities. It might also allow age-friendly agendas to engage more with broader social and political movements such as those around racial, environmental, and intergenerational justice. The impact on older people themselves would ultimately be to help center the less-often-heard voices within aging populations by drawing attention to the different forms of exclusion and inequality experienced in later life. In short, a critical conceptualization of place that recognizes how inequalities shape the places where older people live, will better position aging-in-place researchers to grasp the inequalities facing aging populations.

## Data Availability

This study does not report data and therefore the preregistration and data availability requirements are not applicable.
